# Research on the hydraulic support face guard mechanism and coupling characteristic of rib spalling in large mining heights

**DOI:** 10.1038/s41598-024-58542-5

**Published:** 2024-04-06

**Authors:** Qingliang Zeng, Xiaoqi Ma, Lirong Wan, Yanpeng Zhu, Yanping Yue

**Affiliations:** College of Mechanical and Electronic Engineering, Shandong University of Science and Technology, Qingdao, 266590 China

**Keywords:** Mechanical engineering, Coal

## Abstract

In view of the problem of poor coupling adaptability and easy rib spalling of coal wall in large mining height comprehensive mining, based on the effective inhibition effect of face guard mechanism on coal wall spalling, the structural characteristics and bearing capacity of different structural forms of the face guard mechanism are compared and analyzed. According to the surrounding rock adaptability of the face guard mechanism, established a numerical analysis model for rigid-flexible coupling of the face guard mechanism under different spalling forms. In order to accurately simulate the stress state of the protective mechanism, a variable stiffness spring damping system is used to replace the hydraulic cylinder. The load-bearing performance and response characteristics of the face guard mechanism under rib spalling coupling conditions were analyzed by applying uniform normal load and impact load to the face guard. The findings indicated that, the integral-type face guard mechanism has a better effect on suppressing rib spalling. When the face guard mechanism bears the static load of the coal wall, the entire response process of the face guard jack can be divided into three stages: initial support, increasing resistance bearing, constant resistance bearing; both the impact load position and the coupling state of the rib spalling will affect the characteristics of force transmission at the face guard mechanism’s hinge point, the hinge point between the extensible canopy and the primary face guard is most sensitive to biased load. The research results can provide reference for optimizing the face guard mechanism of large mining height hydraulic support and improving the reliability of coal wall support.

## Introduction

Large mining height comprehensive mining is currently the primary mining way for hard and solid coal seams, it offers advantages such as high production efficiency, reduced pollution in the working face coal seam, and favorable economic returns^1,2^. However, in the wake of the mining height increases, an increase in the exposed area of the rib leads to decreased self-stability, strong dynamic rock pressure impact can easily result in safety issues like rib spalling, roof fall, and support tilting^3–5^. However, due to the suddenness, randomness and complexity of coal wall spalling, it poses a great threat to the safety of comprehensive mining equipment and workers. In addition, after the spalling of the coal wall, the reliability and adaptability of the hydraulic support-surrounding rock coupling support system are reduced, which is easy to cause damage to the support components and unable to achieve efficient production. Therefore, the control of coal wall spalling remains an important challenge that urgently needs to be solved in the development of comprehensive mining technology with high mining height^6,7^.

The occurrence of rib spalling is primarily associated with factors such as coal strength^8^, mining face height^9^, hydraulic support working resistance^10^, and the supporting role of the face guard^11^. Hydraulic supports as the core equipment for controlling the rib fixity, have important functions such as supporting the roof, protecting the coal wall, and isolating the falling of goaf’s rock refuse^12^. The structural features and working rationality of their face guard mechanism are important basic guarantee for the reliability and high adaptability of the hydraulic support-coal wall coupling support system^13^. Singh et al.^14,15^ investigated the influencing factors of rock failure and hydraulic pressure bracket load-bearing performance employing numerical simulation methods, they proposed a prediction method for progressive rock collapse behavior and optimal support capacity. In-depth research on the forms of coal wall instability was conducted by Bai et al.^16,17^ using a strain-softening constitutive model, they observed that shear and tensile failures often occur in mining faces and believed that the mining face shutdown time has a significant impact on rib instability. A model of shear failure for mining faces was established by Wang et al.^18^and the influence factors on the stabilization of the mining face was systematically studied. They pointed out that improving the support capacity of the mining face and the cohesion of the coal mass is the key to improving the rib stability. According to the failure mechanism of coal wall, Kong et al.^19,20^ introduced the " manila + grouting " reinforcement technology to prevent and control coal wall failure. And by combining simulation analysis with on-site application, it was verified that this technology has a significant inhibitory effect on rib spalling. Guo et al.^21^ utilized a “hydraulic support-wall rock” mechanics model to obtain an expression for interaction between the support strength and depth of rib spalling. They observed that as the support strength increased, the depth of rib spalling decreased in a hyperbola curve. Yu et al.^22^ analyzed the influence of coal mass crack distribution on stability of coal wall and the degree of failure under different fracture trends, they concluded that the existence of cracks can induce anisotropic in coal wall stress and displacement. When the face guard direction is perpendicular to the crack surface direction, it is beneficial for improving coal wall stability. Murmu et al.^23^ established a regression model for predicting rib spalling using the methods of stepwise regression and variance analysis, they conducted a parametric analysis on 13 factors affecting rib spalling. The final research showed that rib spalling is highly sensitive to mining-induced stress. Pang et al.^24^ believed that rib spalling can be divided into two stages: the transition from elastic coal wall to the plastic state, and the sliding instability of the plastic coal wall. By establishing a mechanical model of coal wall " fracture-slide", they developed a dual-factor control calculation method for critical support force and hydraulic support working resistance. Zhang et al.^25^ proposed multi-dimensional measures for coal wall stability protection based on the hydraulic support and the "π"-shape mechanical model of coal wall, such as increasing the protective area and protective force, as well as improving the liquid supply speed. The protective capability of the face guard mechanism was analyzed. It is believed that the integrated structure is beneficial for expanding the protective area of coal wall and suppressing coal wall instability. Behera et al.^26^ utilized numerical simulation methods to investigate stress distribution, support load, and failure characteristics of coal walls, they derived a discriminant criterion for critical stress and spalling area of the coal wall. Wang et al.^27^ determined support control parameters and coal wall protection structures based on the three-dimensional dynamic parameters of hydraulic support-coal wall coupling, they suggested that an integrated secondary face guard structure can meet the stability control requirements of coal wall. Yang et al.^28^ conducted research on the recognition of fully-mechanized mining faces and the early warning of underground abnormal situations, this effectively addressed the issue of abnormal detection of the support status of hydraulic support face guard. Wang et al.^29^ used geological radar to detect coal wall cracks and emphasized that the support rigidity of hydraulic support has a significant impacts on the coal wall stability.

Scholars have researched a lot on the failure mode of coal wall, the influence factors of rib spalling, and the coupling theory of support and surrounding rock. However, most of the existing researches are based on coal wall, and there is relatively little research on the support theory of the face guard mechanism, making it difficult to choose the appropriate form of face guard structure, which leads to safety risks and affects the supporting efficiency. Besides, changes in the coupling state will cause damage to hydraulic support components. However, existing literature mostly studies the coal wall and hydraulic support separately, and assumes a single idealization of the coupling relationship between the face guard mechanism and the coal wall. In the actual process of coal mining, coal wall failure and spalling feature are different, and the coupling state between the face guard mechanism and the coal wall is diverse. This coupling relationship and its effects directly impact the stability of the coal wall and the reliability of support systems. Currently, there is a dearth of research on the coupling characteristics between the coal wall and the face guard mechanism. Therefore, considering that rib spalling frequently takes place in the upper and middle coal seam^30,31^, based on the ZZ18000/33/72D four-column support shield hydraulic support, this article aims to establish mechanical models for both integral-type and split-type face guard mechanism of the ZZ18000/33/72D hydraulic support, and compare and analyze the load-bearing disparities between these two structural forms of face guard mechanism. Additionally, based on prevalent rib spalling forms, a rigid-flexible coupling model between the coal wall and the rib mechanism under various spalling forms is established, the force transmission characteristics of pivotal hinge points and the load-bearing response of the face guard mechanism under the various coupling states of rib spalling are explored. This research has significant theoretical support and practical significance for enhancing the reliability of coal wall support in engineering applications.

## Methods

### Structural analysis of the face guard mechanism

**Figure 1:**
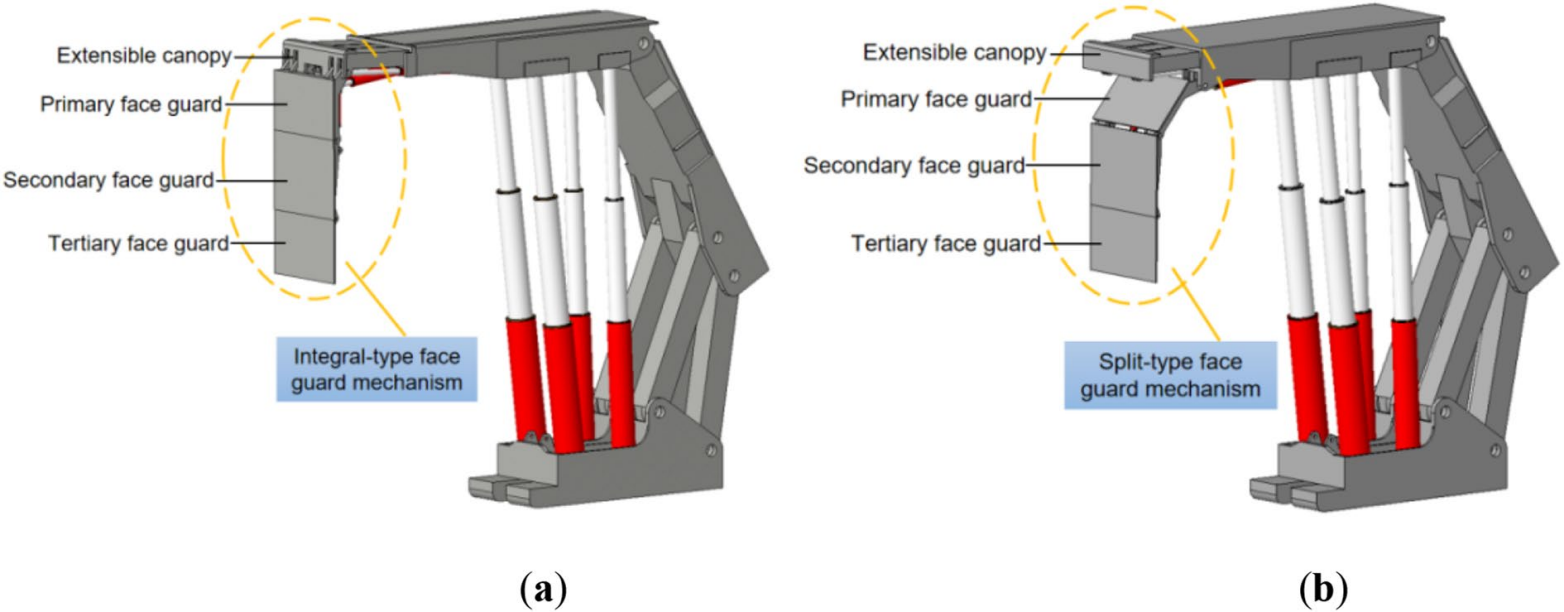
The hydraulic support’s face guard mechanism for large mining height: (**a**) Integral-type face guard mechanism hydraulic support; (**b**) Split-type face guard mechanism hydraulic support.

There are various forms of hydraulic support face guard mechanisms applied to large mining height, and they can be classified into integral-type face guard mechanism and split-type face guard mechanism based on different hinge forms of extensible canopies and face guard^32^, as illustrated in Fig. 1. Although the functions of the face guard mechanisms under different structural forms are similar, there are certain differences in their load-bearing characteristics, and mechanical performance analysis and comparison are needed. Owing to the complexity and variability of the coal wall situation, accurately solving it becomes challenging. In this paper, the force exerted by the coal wall on the face guard is simplified to a concentrated force in order to facilitate analysis. Furthermore, because of the most frequent occurrence of coal wall spalling above the middle of the coal seam^33,34^, only the primary and secondary face guards play a crucial role in maintaining the coal wall stability, the tertiary face guard have a large moment arm but exert relatively small force, allowing us to disregard their bearing capacity on the coal wall. Hence, the force analysis needs to focus solely on the face guards of primary and secondary in two structures.

### Theoretical model of the integral-type face guard mechanism

**Figure 2:**
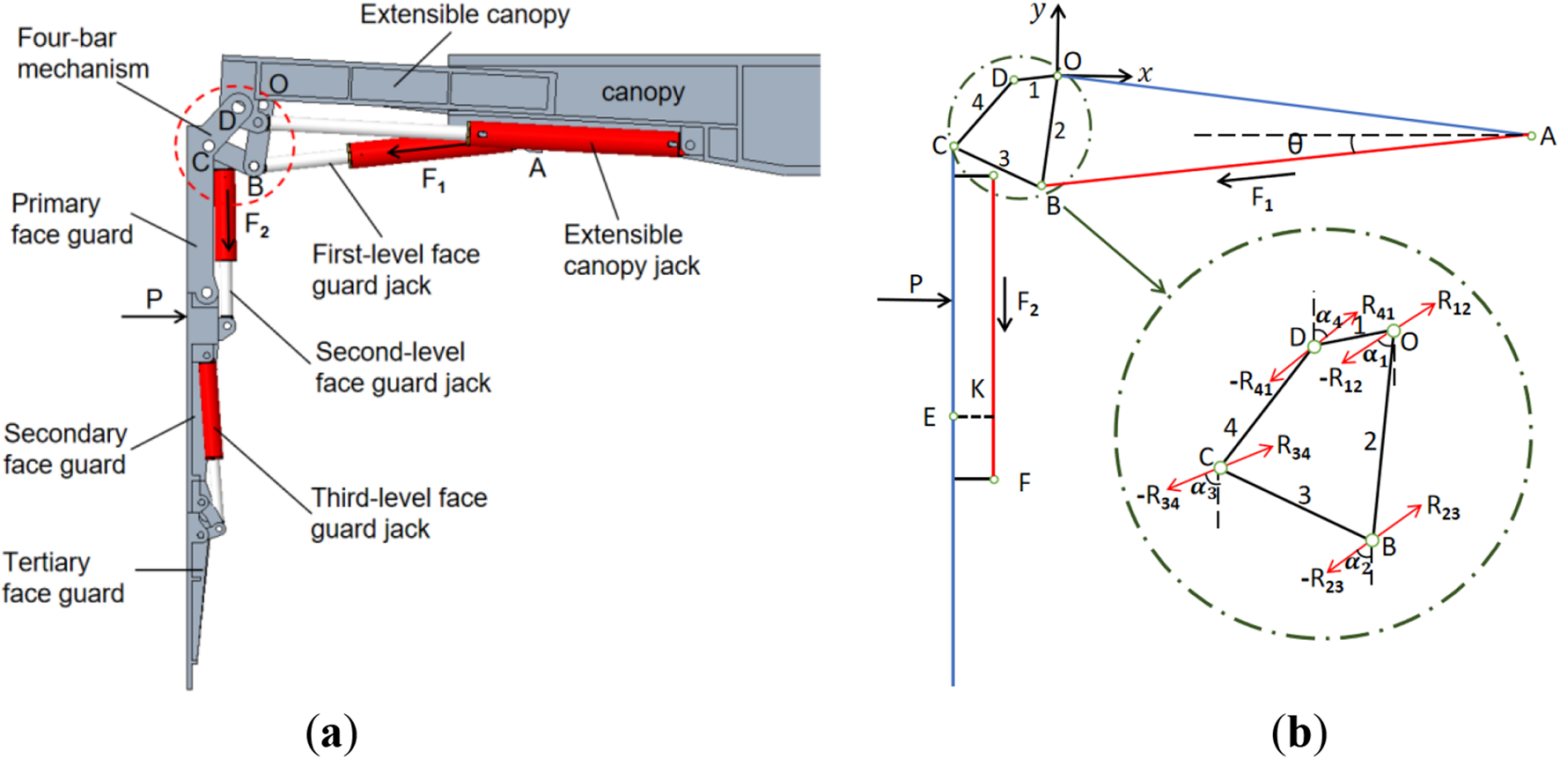
The Structure and force analysis of the integral-type face guard mechanism: (**a**) structure style diagram of the integral-type face guard mechanism; (**b**) force Analysis diagram of the integral-type face guard mechanism.

The structural style that unites the extendable canopy with the face guard is known as an integral-type face guard mechanism. The connection relationships among each structure are depicted in Fig. 2a. Created a rectangular coordinate system, with rod 2 and the extensible canopy’s hinge point serving as the origin (O). rod 1 (OD), rod 2 (OB), rod 3 (BC), and rod 4 (CD) form a four-bar linkage. P represents the concentrated force exerted by the coal wall on the face guard; The force generated by the first-level face guard jack acting on the hinge point B is denoted as *F*_*1*_, and the force generated by the second-level face guard jack acting on the hinge point F is denoted as *F*_*2*_. The angle between the first-level face guard jack and the x-axis is recorded as $\theta$, and K is the distance from the hinge point E to the second-level face guard jack. *R*_*ij*_ represents the force exerted by rod i on rod j in a four-bar linkage, where both i and j take values of 1, 2, 3, and 4, the force exerted by rod j on rod i is *R*_*ji*_, and *R*_*i*j_ = − *R*_*ji*_. As illustrated in Fig. 2b, performing a force analysis on face guard mechanism of the integral-type, and list the corresponding force and torque equilibrium equations as follows:

Force analysis of rod 2:

1 $$\left\{ {\begin{array}{*{20}c} { - l_{{OB}} \;\;cos\;\;\angle OB \cdot R_{{23}} \;\;sin\;\;\alpha _{2} \;\; + \;\;l_{{OB}} \;\;sin\;\;\angle OB \cdot R_{{23}} \;\;cos\;\;\alpha _{2} \;\; = } \\ { - l_{{OB}} \;\;cos\;\;\angle OB \cdot F\;\;{\text{cos}}\;\;\theta \;\; + \;\;l_{{OB}} \;\;sin\;\;\angle OB \cdot F\;\;sin\;\;\theta } \\ {R_{{12}} \;\;sin\;\;\alpha _{1} - R_{{23}}\; sin\;\;\alpha _{2} = - F\;\;cos\;\;\theta } \\ {R_{{12}} \;\;cos\;\;\alpha _{1} - R_{{23}} \;\;cos\;\;\alpha _{2} = - F\;\;sin\;\;\theta } \\ \end{array} } \right.$$

Force analysis of rod 3:

2 $$\left\{ {\begin{array}{*{20}c} {l_{{BC}} \;\;cos\;\;\angle BC \cdot R_{{34}} \;\;sin\;\;\alpha _{3} \;\; + \;\;l_{{BC}} \;\;sin\;\;\angle BC \cdot R_{{34}}\; cos\;\;\alpha _{3} \;\; = \;\;0} \\ {R_{{23}} \;\;sin\;\;\alpha _{2} - R_{{34}} \;\;sin\;\;\alpha _{3} = 0} \\ {R_{{23}} \;\;cos\;\;\alpha _{2} - R_{{34}} \;\;cos\;\;\alpha _{3} = 0} \\ \end{array} } \right.$$

Force analysis of rod 4:

3 $$\left\{ {\begin{array}{*{20}c} {l_{{CD}} \;\;cos\;\;\angle CD \cdot R_{{41}} \;\;sin\;\;\alpha _{4} - l_{{CD}} \;\;sin\;\;\angle CD \cdot R_{{41}} \;\;cos\;\;\alpha _{4} \;\; + \;\;y_{p} \cdot P = 0} \\ {R_{{34}} \;\;sin\;\;\alpha _{3} - R_{{41}} \;\;sin\;\;\alpha _{4} + \;\;P\;\; = \;\;0} \\ {R_{{34}} \;\;cos\;\;\alpha _{3} - R_{{41}} \;\;cos\;\;\alpha _{4} \;\; = \;\;0} \\ \end{array} } \right.$$

Among them, ${l}_{XY}$ is the length of the rod XY, $\angle XY$ is the angle between the rod XY and the vertical direction, ${\alpha }_{i}$ is the angle between the ${R}_{i}$ and the vertical direction, ${y}_{p}$ is the distance between the origin O and the coal wall load P.

Due to the complexity of using analytical methods to solve the force equations of a four-bar linkage, for the convenience of calculation and accuracy of results, MATLAB software is used for automatic solution. Therefore, the above equilibrium equations are expressed in matrix form as follows:

4 $$\left[\begin{array}{ccccccccc}0& 0& {-l}_{OB}\;\;{cos}\;\;\angle OB \;\; & {l}_{OB}\;\;{sin}\;\;\angle OB& 0& 0& 0& 0& 0\\ 1& 0& -1& 0& 0& 0& 0& 0& 0\\ 0& 1& 0& -1& 0& 0& 0& 0& 0\\ 0& 0& 0& 0& {l}_{BC}\;\;{cos}\;\;\angle BC& {l}_{BC}\;\;sin\;\angle BC& 0& 0& 0\\ 0& 0& 1& 0& -1& 0& 0& 0& 0\\ 0& 0& 0& 1& 0& -1& 0& 0& 0\\ 0& 0& 0& 0& 0& 0& {l}_{CD}\;\;{cos}\;\;\angle CD& -{l}_{CD}\;\;{sin}\;\;\angle CD& {y}_{p}\\ 0& 0& 0& 0& 1& 0& -1& 0& 1\\ 0& 0& 0& 0& 0& 1& 0& -1& 0\end{array}\right]\left[\begin{array}{c}{R}_{12}\;\;{sin}\;\;{\alpha }_{1}\\ {R}_{12}\;\;{cos}\;\;{\alpha }_{1}\\ {R}_{23}\;\;{sin}\;\;{\alpha }_{2}\\ {R}_{23}\;\;{cos}\;\;{\alpha }_{2}\\ {R}_{34}\;{sin}\;\;{\alpha }_{3}\\ {R}_{34}\;\;{cos}\;\;{\alpha }_{3}\\ {R}_{41}\;{sin}\;\;{\alpha }_{4}\\ {R}_{41}\;\;{cos}\;\;{\alpha }_{4}\\ P\end{array}\right]\left[\begin{array}{ccccccccc}{-l}_{OB}\;\;{cos}\;\;{\angle }OB& {l}_{OB}\;\;{sin}\;{\angle }OB& 0& 0& 0& 0& 0& 0& 0\\ -1& 0& 0& 0& 0& 0& 0& 0& 0\\ 0& -1& 0& 0& 0& 0& 0& 0& 0\\ 0& 0& 0& 0& 0& 0& 0& 0& 0\\ 0& 0& 0& 0& 0& 0& 0& 0& 0\\ 0& 0& 0& 0& 0& 0& 0& 0& 0\\ 0& 0& 0& 0& 0& 0& 0& 0& 0\\ 0& 0& 0& 0& 1& 0& 0& 0& 0\\ 0& 0& 0& 0& 0& 1& 0& 0& 0\end{array}\right]\left[\begin{array}{c}F\;{cos}\;\theta \\ F\;{sin}\;\;\theta \\ 0\\ 0\\ 0\\ 0\\ 0\\ 0\\ 0\end{array}\right]$$

When the coal wall load P acts on the integral-type face guard mechanism and only the first-level face guard jack is loaded, the calculation formula is as shown above, and the bearing value is denoted as ${{\text{P}}}_{1}$. When the coal wall load P acts on the second-level face guard jack, the face guard jacks of first and second level are compressed simultaneously. At this time, the ultimate load-bearing capacity of the second level face guard jack is necessary to be calculated, and the integral-type face guard mechanism’s bearing capacity should be comprehensively taken into account^35,36^. Determine and calculate using the following equation:

5 $$\left\{ {\begin{array}{*{20}l} {P = P_{1} {\text{ }}} \hfill & {P_{1} \cdot y_{d} - F_{2} \cdot K \le 0} \hfill \\ {P \cdot y_{d} = F_{2} \cdot K{\text{ }}} \hfill & {P_{1} \cdot y_{d} - F_{2} \cdot K{ > }0{\text{ }}} \hfill \\ \end{array} } \right.$$

In the equation,${y}_{d}$ is the distance between the coal wall load P and the hinge point E.

**Figure 3:**
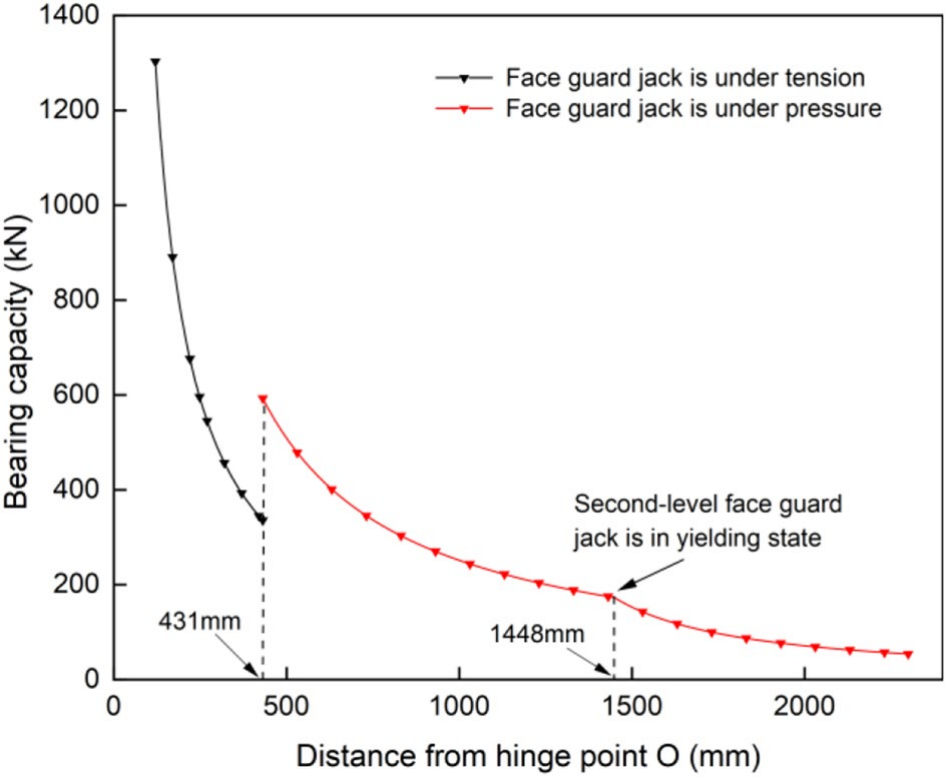
Bearing capacity diagram of integral-type face guard mechanism.

Based on the structural size parameters of the integral-type face guard, ${l}_{OB}$=350 mm, ${l}_{OD}$=140.4 mm, ${l}_{CD}$=279.9 mm, ${l}_{BC}$=300.5 mm, $\angle OB$=8.5, $\angle OD$=84.8°, $\angle CD$=41.4°, $\angle BC$=65.8°, $\theta$=7.9°, 120 mm $<{y}_{p}<$ 2300 mm, *K* = 112 mm. The primary face guard adopts two double extensible jacks with a cylinder diameter of 125 mm and a working resistance of 466 kN / 298 kN. The secondary face guard adopts two double extensible jacks with a cylinder diameter of 100 mm and a working resistance of 298 kN. As a result, when under pressure, the first-level face guard jack can withstand a maximum force of 932 kN, and when under tension, it can withstand a maximum force of 528 kN. The maximum force that the second-level face guard jack can withstand under compression is 596 kN. To obtained the integral-type face guard mechanism’s load-bearing characteristic curve, substitute the above parameters into Eqs. (4) and (5). The result is depicted in Fig. 3.

### Theoretical model of the split-type face guard mechanism

**Figure 4:**
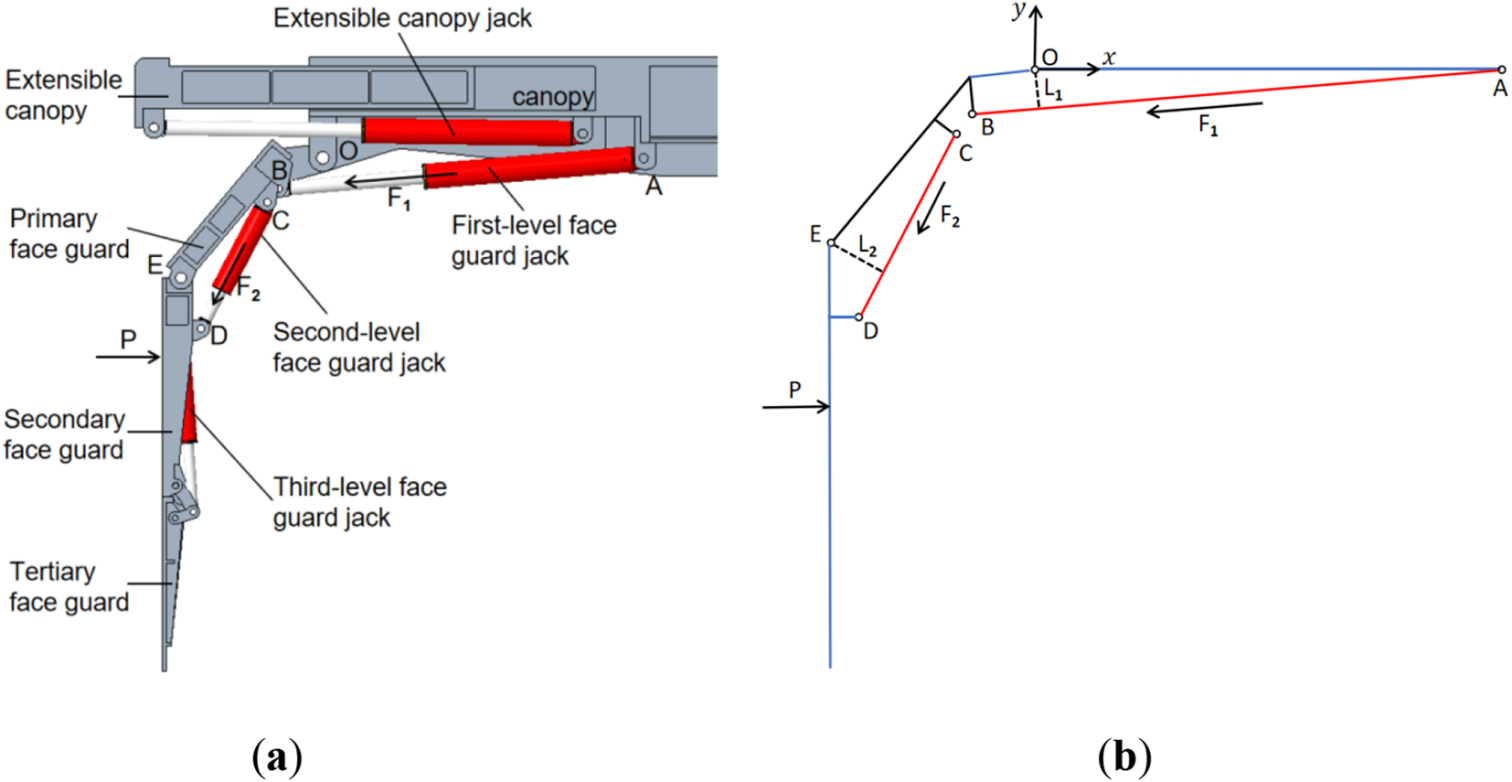
The structure and force analysis of the split-type face guard mechanism: (**a**) structure diagram of the split-type face guard mechanism; (**b**) force Analysis diagram of the split-type face guard mechanism.

The split-type face guard mechanism is a structural form in which the face guard is hinged with the canopy and designed separately from the extensible canopy. The connection relationship between each structure is shown in Fig. 4a. Establish an rectangular coordinate system using the hinge point O between the canopy and the primary face guard as coordinate origin. The forces generated by the first-level face guard jack on hinge point B and the second-level face guard jack on hinge point D are denoted as *F*_*1*_ and *F*_*2*_, respectively. The primary face guard is not in touch with the coal wall when the split-type face guard mechanism is employed for support, the secondary face guard is subject to the coal wall load P. The entire stress analysis process is relatively simple, as shown in Fig. 4b, but it is still necessary to consider the yield limit of the first and second level face guard jacks simultaneously. The following is the equilibrium equation for force analysis:

6 $${F}_{1}\cdot {L}_{1}={P}_{1}\cdot y$$

7 $$\left\{ {\begin{array}{*{20}l} {P = P_{1} } \hfill & {P_{1} \cdot y - F_{2} \cdot L_{2} \le 0} \hfill \\ {P \cdot y = F_{2} \cdot L_{2} } \hfill & {P_{1} \cdot y - F_{2} \cdot L_{2} { > }0} \hfill \\ \end{array} } \right.$$

Among them, P_1_ is the bearing value of the split-type face guard mechanism with only the first-level face guard jack under pressure, the vertical distance from the coordinate origin O to the first-level face guard jack is called ${L}_{1}$, and the vertical distance between the hinge point E and the second-level face guard jack is called ${L}_{2}$, and $y$ is the distance between the coal wall load P and the coordinate origin O.

**Figure 5:**
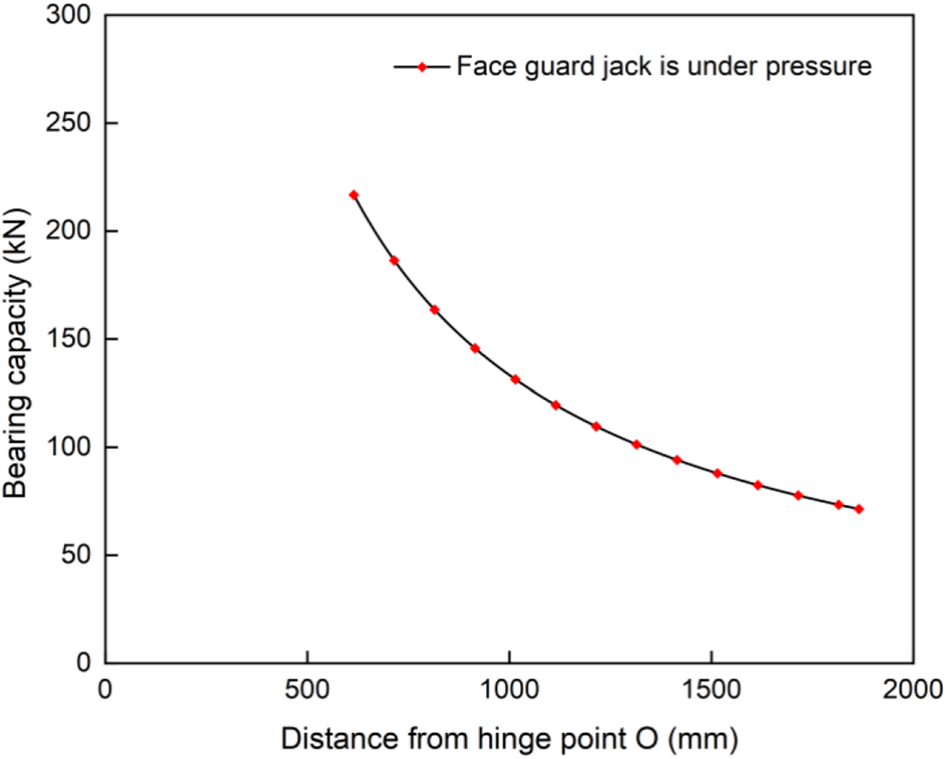
Load-bearing capacity diagram of split-type face guard mechanism.

Refer to the structural size data of the split-type face guard mechanism, ${L}_{1}$ =136 mm, ${L}_{2}$=249 mm, 615 mm < y < 1865 mm. For the purpose of accurately comparing the load-bearing properties of two structural forms of the face guard mechanism, the same first and second level face guard jacks were used. Therefore, ${F}_{1}$=932 kN, ${F}_{2}$=596 kN (when the split-type face guard mechanism is used for support, due to its structural characteristics, it is only under pressure). By incorporating the above parameters into the balance equation, the bearing characteristic curve of the split-type face guard can be obtained, as shown in Fig. 5.

### Numerical model of coupling of the coal wall structure and the integral-type face guard mechanism

The coal wall stability is directly impacted by the coupling relationship between the face guard mechanism and the coal wall structure^37,38^. On the one hand, the face guard mechanism can apply actively support to the rib, avoid spalling coal blocks and gangue from damaging workers and equipment. On the other hand, it can prevent progressive damage to the coal wall and the further expansion of the rib spalling range, thereby inducing a larger zone of surrounding rock instability. Due to its four-bar articulated structure, the integral-type face guard mechanism moves simultaneously with the extensible canopy, offering high flexibility and strong adaptability to the surrounding rock under different forms of spalling. Therefore, establish numerical model of coupling of the coal wall structure and the integral-type face guard mechanism.

During the support process of the hydraulic support face guard mechanism, the length of the emulsion in its hydraulic cylinder is constantly changing due to the influence of coal wall load, resulting in a continuous dynamic change in the stiffness of the face guard mechanism jack. For an accurate simulation of the stiffness characteristics of the hydraulic support face guard mechanism, a variable stiffness spring damping system is employed as an equivalent replacement. The formula for determining the sealed emulsion’s equivalent stiffness in the hydraulic cylinder is given in Eq. (8):

8 $$K = \frac{2S\delta E}{{S + 2LG\delta E}}$$

In the formula, K represents the equivalent stiffness coefficient of the hydraulic cylinder, kN/mm; G is the bulk elastic modulus of the emulsion, G = 1.95 × 10^3^ MPa; E is the elastic modulus of the cylinder block, E = 2.1 × 10^5^ MPa; S is the hydraulic cylinder’s effective working area, m^2^; δ is the wall thickness of the hydraulic cylinder, mm; L is the effective liquid column length of the hydraulic cylinder, mm. According to the working principle and stiffness calculation method of the jack, the variable stiffness curve of the jack is obtained by analyzing the stiffness characteristics of the jack under different lengths, and the curve is endowed to the equivalent spring damping system.

**Figure 6:**
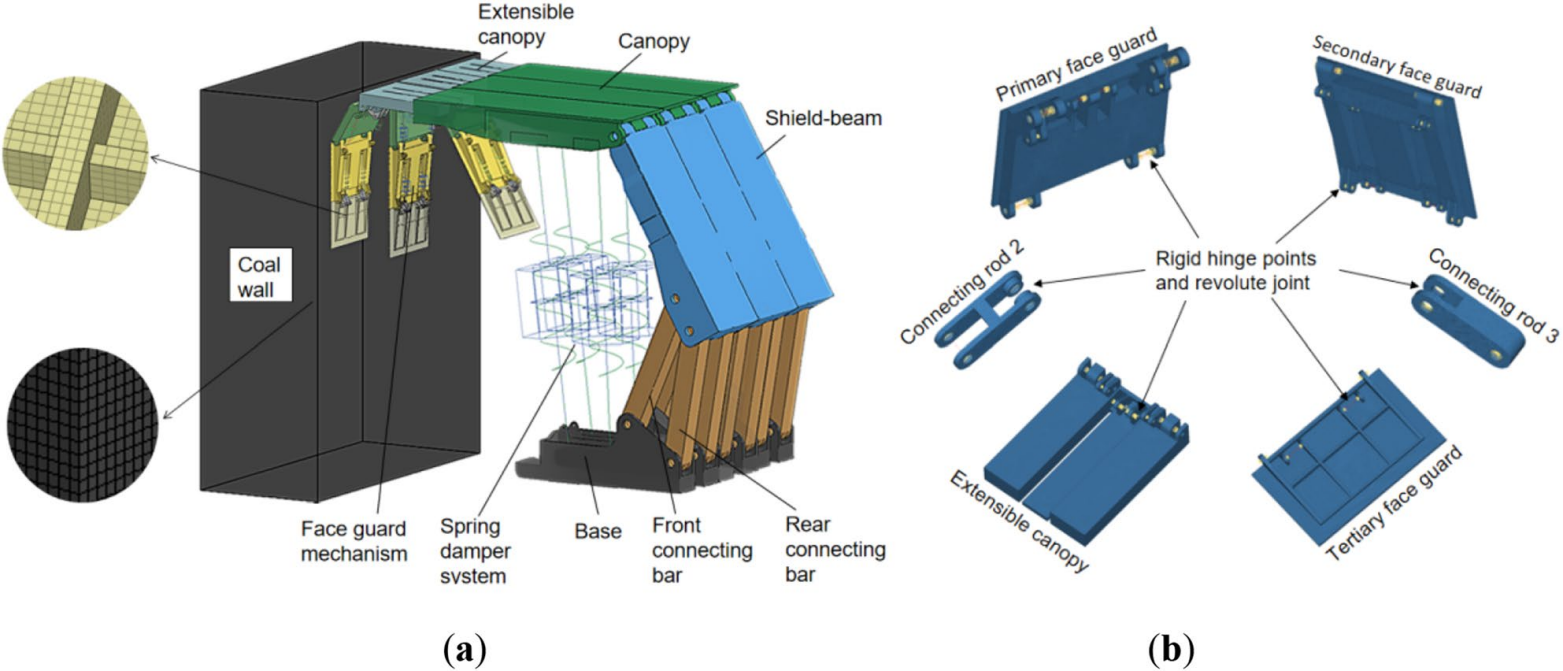
Rigid-flexible coupling model of large mining height hydraulic support: (**a**)rigid-flexible coupling model of hydraulic support-coal wall; (**b**) rigid-flexible coupling model of face guard mechanism.

Through Hypermesh finite element software, the three-dimensional models of large mining height hydraulic supports under different working conditions are meshed. Rigid areas are established with rotational pairs by setting up component hinge points, and a friction coefficient of 0.3 is defined. Spring and damping elements are then introduced in the finite element analysis software LS PrePost, variable stiffness springs replace hydraulic cylinders of hydraulic supports, and various settings are defined for numerical simulation. Additionally, all degrees of freedom of the lowest grid nodes of the base are constrained to simulate the fixed support of the hydraulic support. Each structure of the hydraulic support is welded with high-strength alloy structural steel plates (Q550, Q690, Q890) of different strengths. Relevant materials are defined by setting parameters such as elastic modulus, density, yield strength, Poisson’s ratio, etc. The material property parameters are shown in Table 1. Figure 6 illustrates the rigid-flexible coupling model of the hydraulic support for large mining heights, established based on the aforementioned assumptions and settings.

**Table 1:** Material property parameters of hydraulic support.

Material type	Density /kg∙m^−3^	Elastic modulus /GPa	Poisson’s ratio	Yield strength /MPa
Q550	7850	210	0.3	550
Q690	7850	210	0.3	690
Q890	7850	210	0.3	890

### Numerical simulation reliability verification

**Figure 7:**
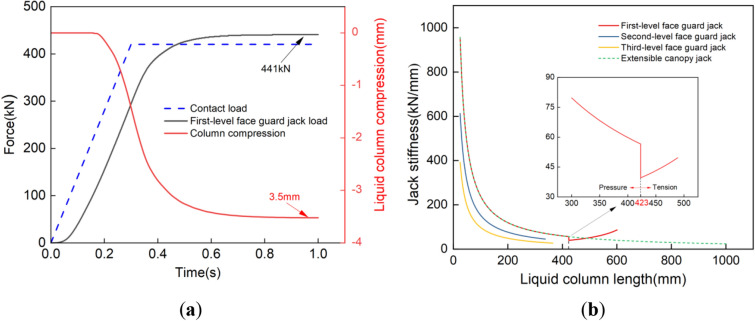
Numerical simulation reliability verification: (**a**) steady state load response curve of the face guard mechanism; (**b**) jack stiffness of the face guard mechanism.

Perform steady-state loading and stiffness analysis on the face guard mechanism, evaluate the reliability of numerical simulation for face guard mechanism by testing the steady-state response performance and equivalent substitution effect of each structure in the numerical model. Considering that the resistance borne by the first-level face guard jack during operation, as studied in this article, is 466 kN. To simulate the contact loading effect between the face guard and the coal wall, a vertical uniform load with a total load less than the working resistance was applied to all grid nodes of the primary face guard, the contact load was set at 420 kN and the static load time was 0.3 s. The steady-state response curve of the face guard bearing is shown in Fig. 7a. Because the spring preload serves as an equivalent substitute for the initial support force of the face guard, the first-level face guard jack is not compressed when the contact load is less than the initial support force of the face guard in 0–0.2 s. As the contact load continues to increase and exceeds the initial support force of the face guard, the First-level face guard jack gradually starts compressing. Upon reaching a stabilized external load, the first-level face guard jack settles at a load of 441 kN, the liquid column compression is 3.5 mm, and the safety valve remains closed. During the steady-state loading process, the mechanical response and liquid column change trend of the hydraulic support face guard mechanism are consistent with the actual action process of the support, and the simulation model has good reliability.

Table 2 lists the main parameters of the jack for the face guard mechanism. Incorporating the data from Table 2 into Eq. (8) and introducing the safety valve overflow cutoff process yield the stiffness change curve of the face guard mechanism jack, depicted in Fig. 7b. This chart shows that the extensible canopy jack, the second and third level face guard jack are only under pressure during the bearing stage of the face guard, while the first-level face guard jack is in a tensile state when the liquid column length is less than 423 mm, and in a compressive state when the liquid column length is greater than 423 mm. Its equivalent replacement effect is consistent with the actual bearing situation of the hydraulic cylinder, which can ensure the accuracy of the analysis.

**Table 2:** Main parameters of the face guard mechanism jack.

	Cylinder diameter /mm	Cylinder wall thickness /mm	Rod diameter /mm	Effective liquid column length /mm
Extensible canopy jack	125	10.5	90	1023
First-level face guard jack	125	10.5	90	692
Second-level face guard jack	100	10.5	70	338
Third-level face guard jack	80	11	60	365

### Ethics approval

The authors warrant that the work has not been published before in any form and that the work is not concurrently submitted to another publication. The authors also warrant that the work does not libel anyone, infringe anyone’s copyright, or otherwise violate anyone’s statutory or common law rights.

## Results and discussion

### Comprehensive comparison of face guard mechanism under different structural forms

**Figure 8:**
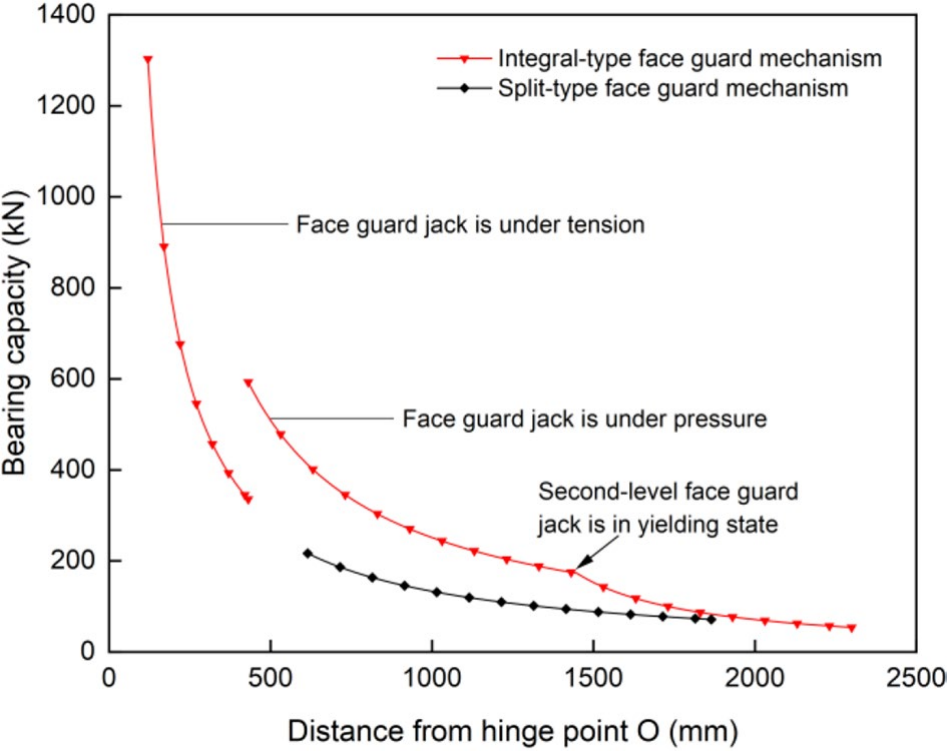
Bearing capacity diagram of face guard mechanism under two structural forms.

Figure 8 illustrates the bearing capacity of various structural forms of face guard mechanism. During the process of supporting the coal wall with an integral-type face guard mechanism, when there is 431 mm between the coal wall load position and the hinge point O, for the first-level face guard jack, it is at the tension and compression balance point and the bearing capacity of the jack in both states decreases as the load position moves downward. The first-level face guard jack has a maximum bearing capacity of 1303 kN under tension and 593 kN under compression. When the coal wall load is applied to secondary face guard, a ’yielding ’ phenomena occurs at the second-level face guard jack, which is located 1448 mm from the point O. The support capability of face guard mechanism is significantly reduced and the maximum protective force at the tail end of the integral-type face guard mechanism amounts to only 54 kN. The load-bearing capacity of the split type face guard mechanism decreases gradually as the coal wall load position descends. Since the primary face guard only contributes to structural transmission and primarily depends on the secondary face guard to bolster the rib, the effective support capacity and support scope of the split-type are small, with a maximum bearing value of 217 kN and a minimum bearing value of 71 kN.

The bearing capacity variation trends of the two structures are similar. However, the integral type’s bearing capacity and effective support range surpass those of the split type. The highest load carrying capacity of integral-type is approximately 6 times that of split type, and the effective support range is about 1.7 times wider. As the coal wall loading position progresses downwards along the face guard, the disparity between two bearing capacities diminishes gradually. Ultimately, both exhibit their weakest bearing capacity at the face guard’s terminus. As a result, during the process of rib spalling, the face guard mechanism needs to be able to respond quickly and provide precise support in order to prevent contact with the face guard’s end. This contact could lead to safety issues such as insufficient support by the hydraulic support, and under the same conditions of large mining height and comprehensive mining, it is preferable to prioritize the use of an integral-type face guard mechanism.

### Coupling characteristics analysis of face guard mechanism and coal wall under different spalling situations

**Figure 9:**
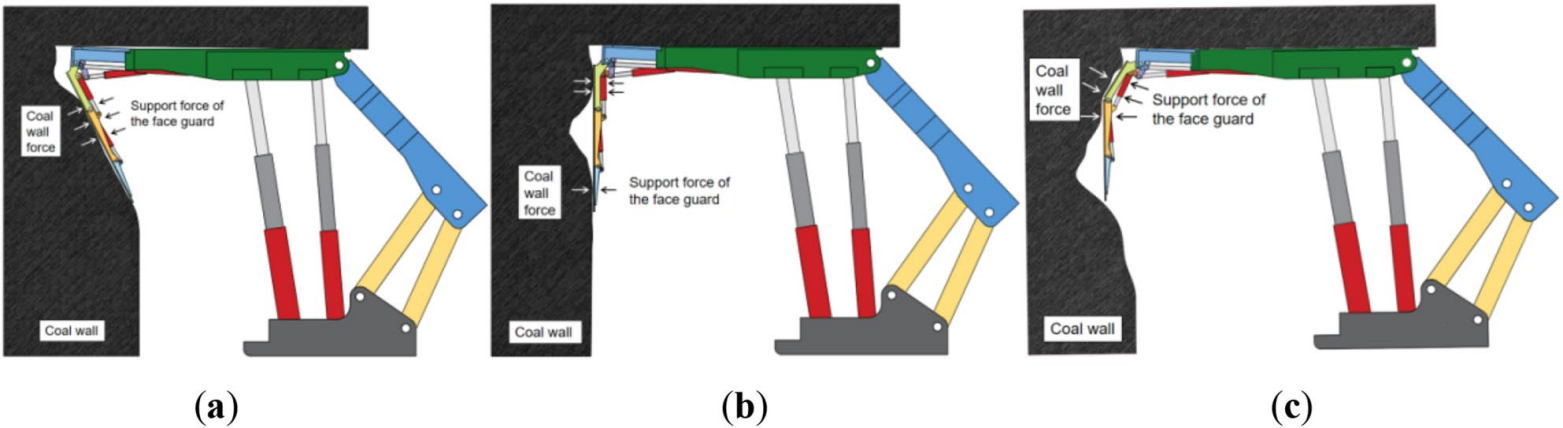
Coupling diagram of the integral-type face guard mechanism-coal wall different rib spalling: (**a**) upper rib spalling; (**b**) middle-upper rib spalling; (**c**) middle rib spalling.

The actual contact condition between the coal wall and the hydraulic support face guard varies because of factors such as damage degree and flatness of coal wall structure of the full-mechanized mining face with a large mining height. However, the integral-type face guard mechanism can match the structural posture of the face guard with the coal wall structure using a four-bar mechanism based on the rib spalling form of the mining face, this maximizes fit closely to the coal wall and achieves the best structural coupling effect, fully utilizing the support function of the face guard mechanism on the coal wall, as depicted in Fig. 9. Figure 9a depicts the upper spall of the coal wall, which is a form of spall that accounts for a large proportion of the full-mechanized mining face with large mining height. Because the junction between the coal wall working face and the roof is susceptible to stress concentration, the degree of pressure concentration rapidly increases as the strong impact rock pressure acts on the roof above the coal wall. When the roof pressure surpasses the ultimate bearing capacity of the coal wall, damage occurs, causing the upper portion of the coal wall to flake. For coal seams with high development of internal fractures and low distribution of mining stress, under the action of mining stress, the fractures fully expand and connect, resulting in small-scale fracture failure type spalling in the upper and middle parts of the coal body, as shown in Fig. 9b. Influenced by the development degree of internal fracture in coal, the sizes of coal blocks resulting from the middle-upper rib spalling vary. While the level of harm is low, the difficulty of control is high. In intact coal seams with a low development of internal fractures and high degree of even distribution of mining stress, when the mining stress exceeds the strength of the coal wall, the coal wall experiences locally concentrated large block size middle spalling, as shown in Fig. 9c, this type of spalling has a high degree of damage.

**Figure 10:**
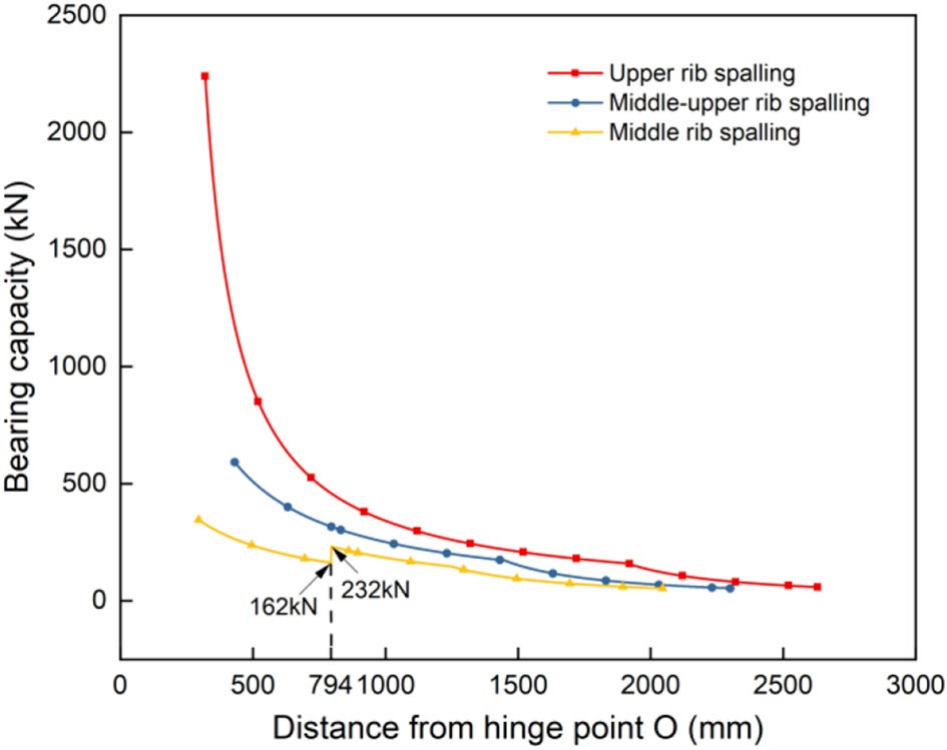
Bearing capacity of the integral-type face guard mechanism under different rib spalling.

Simplify the force exerted by the coal wall on the protective mechanism as a point load, and use the same calculation method as the theoretical model of the integral-type face guard mechanism to analyze the force under different support postures. The load-bearing characteristic curve of the face guard mechanism under various rib spalling forms can be obtained by calculating the force and torque balance equations, illustrated in Fig. 10. Under various spalling conditions, the integral-type face guard mechanisms exhibit a similar bearing capacity trend, where in the overall load-bearing capacity decreases with a downward movement in the coal wall load position. Under the action of a four-bar mechanism, the face guard closely resembles the structure of the coal wall in the form of the middle rib spalling. The direction of the face guard’s support force changes at a distance of 794 mm from hinge point O, causing a sudden increase in the load-bearing capacity from 162 to 232 kN. This indicates that it is advantageous to align the face guard structure’s posture with the coal wall structure in order to improve the coal wall’s stability. The primary face guard of the integral-type face guard mechanism plays a main role in supporting the coal wall. Different forms of spalling result in diverse coupling postures between the coal wall and the primary face guard, leading to significant variations in the structural bearing area’s support capacity. Notably, the face guard mechanism’s maximum bearing capacity under the upper rib spalling form is approximately 3.8 times that of the middle-upper rib spalling, and roughly 6.5 times that of the middle rib spalling. Within the 0.8 m range from the hinge point, when the upper coal wall is spalling, the face guard mechanism’s bearing capacity is noticeably greater than the middle-upper rib spalling’s. The face guard mechanism’s bearing capacity is weakest when the middle coal wall is spalling. Simultaneously, within this range, the face guard mechanism’s bearing capacity during upper rib spalling experiences a rapid decline due to the minimum angle between its connecting rods. Beyond the 0.8 m range from the hinge point, the disparity in bearing capacity among the three gradually decreases, and under the three types of spalling forms, the tail end of face guard mechanism’s load-bearing capacity is relatively weak.

### Static load-bearing response of the face guard mechanism

**Figure 11:**
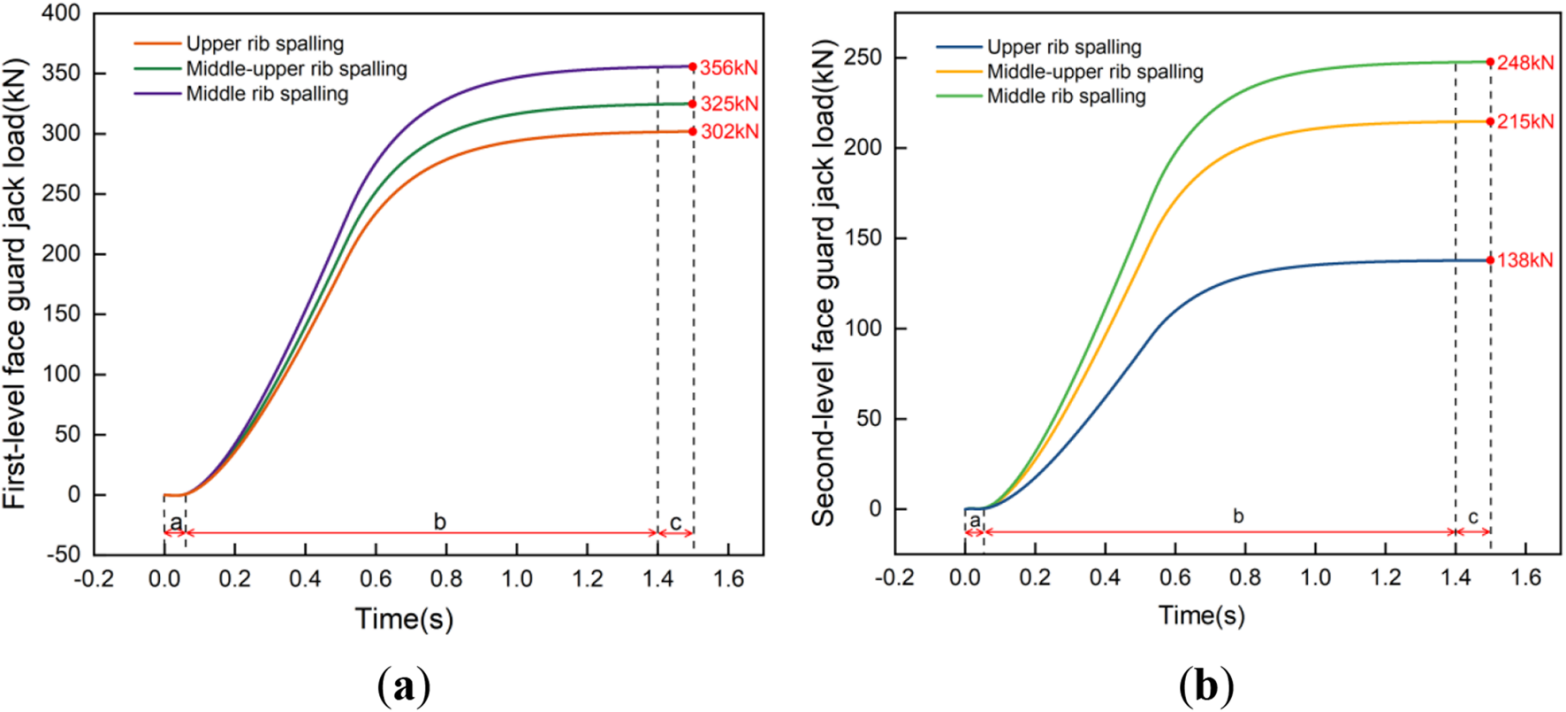
Load response curve of the face guard mechanism: (**a**) load response curve of the first-level face guard jack; (**b**) load response curve of the second-level face guard jack.

**Figure 12:**
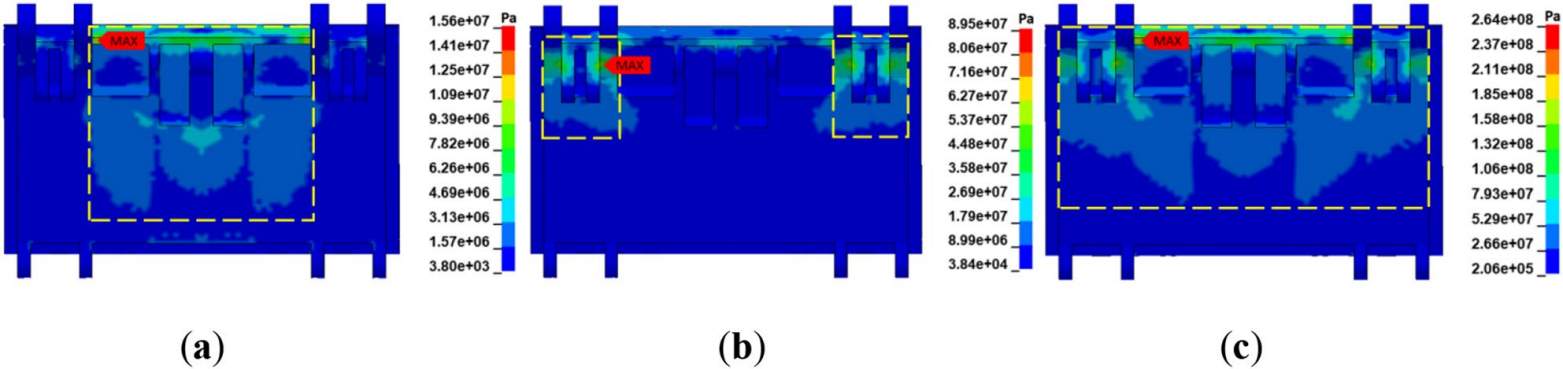
Stress nephogram of the primary face guard: (**a**) initial support; (**b**) increasing resistance bearing; (**c**) constant resistance bearing.

**Figure 13:**
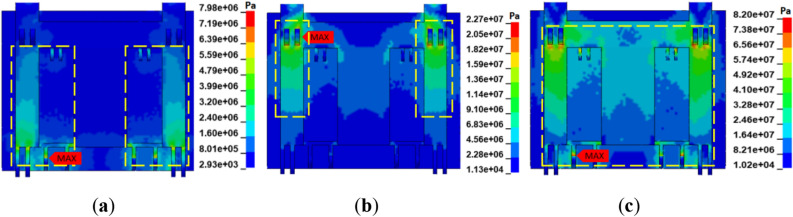
Stress nephogram of the secondary face guard: (**a**) initial support; (**b**) increasing resistance bearing; (**c**) constant resistance bearing.

Based on the established model of the hydraulic support, a uniformly distributed load of equal magnitude was applied on the contact surface of the coal wall and the face guard to explore the adaptability of the face guard mechanism’s static load strength under multiple coupling conditions. To ensure that the jacks at all levels of the face guard mechanism are stable and do not overflow, the static load size of the face guard at all levels is set to be 80 kN. Figure 11 illustrates load-bearing response curve of the face guard jack. The face guard mechanism’s static bearing capacity is divided into three stages based on the trend of load changes of the first and second level jacks: initial support (stage a), increased resistance bearing (stage b), and constant resistance bearing (stage c). and analyzed in conjunction with the stress change cloud charts of the face guard shown in Figs. 12 and Fig. 13. Considering the face guard’s constant stress distribution properties under three coupling states, the adaptability of static load strength was examined using the middle rib spall as a specific example. During the initial support stage, there is less coal wall load than the face guard jack’s active support force, and the face guard jack is not compressed. Stress within the face guard predominantly concentrates in the central region of the primary face guard and the lower part of the tertiary face guard’s rib plate, with the maximum stress occurring at the face guard’s rib plate. In the increasing resistance bearing stage, as the load of the coal wall increases, the load on the face guard jack increases rapidly and tends to slow down. Stress distribution areas of the primary and secondary level face guards shift towards the hinge area between the face guard and the jack, with the maximum stress concentrating at the jack’s hinge hole position. During the constant resistance bearing stage, as the coal wall load tends to stabilize, and the stress distribution area covers the entire face guard, the maximum stress value is once again located at the reinforcement plate of the face guard, and the load on jacks at all levels remains constant. Based on the above analysis, the stress distribution and stress concentration areas of the face guard at different bearing stages are different. High strength plates need to be selected in each stage to meet the static load support requirements of the hydraulic support face guard, and the stress concentration areas should be appropriately strengthened and repaired.

**Figure 14:**
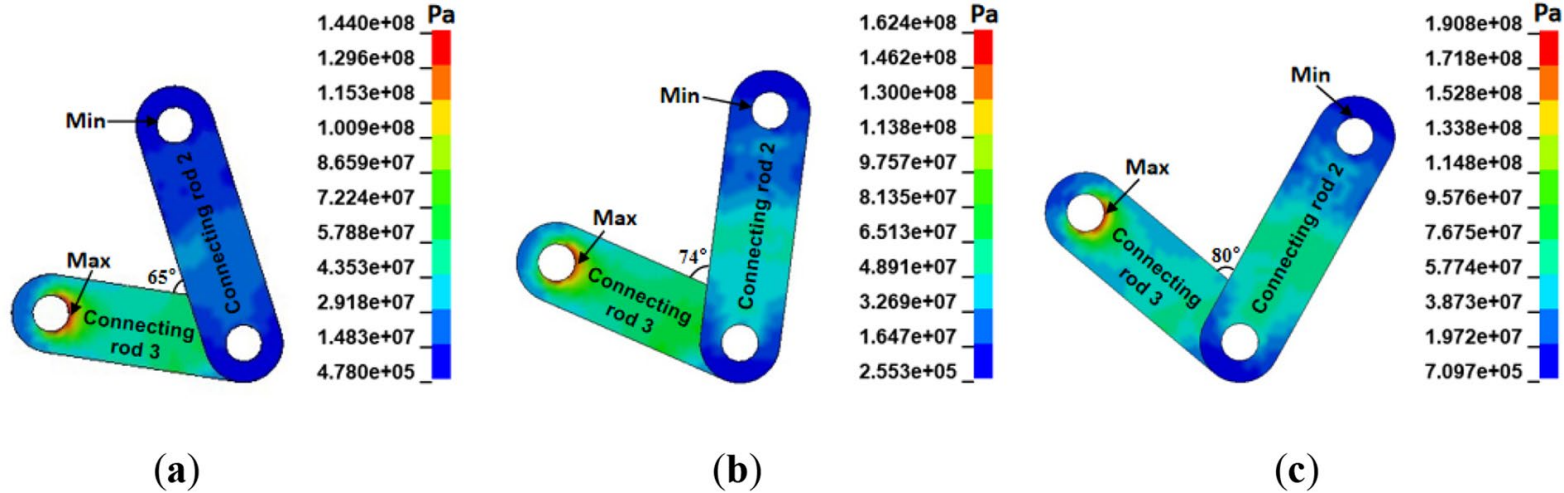
Stress nephogram of connecting rods with different rib spalling: (**a**) upper rib spalling; (**b**) middle-upper rib spalling; (**c**) middle rib spalling.

The integral-type face guard’s four-bar mechanism consists of a primary face guard, an extensible canopy, linkage 2, and linkage 3. It can drive the face guard to adhere as closely as possible to the coal wall in accordance with the rib spalling forms at the mining working face, achieve the best coupling effect between the face guard mechanism and the rib spalling structure, and fully utilize the face guard’s supporting effect on the coal wall. Therefore, an analysis of the adaptability of the static load strength of the four-bar mechanism is necessary. Figure 14 illustrates the stress distribution of the connecting rod under various spall forms. The image shows that, under various spalling situations, the connecting rod’s stress distribution is essentially constant, with the major face guard’s hinge point and linkage 3 experiencing the highest stress. This is because the primary face guard performs its support function by directly contact the coal wall when carrying the load on the coal wall, so stress concentration of the direction of tension is easy when supporting the coal wall with its connectors. The extensible canopy of the integral-type hydraulic support only serves to coordinate the coupling between the coal wall and the face guard, so the minimum stress value is situated above the hinge hole of connecting rod 2 and the extensible canopy. When the middle coal wall is spalling, the average and maximum stress of the connecting rod are the highest, and when the upper coal wall is spalling, they are at their lowest. The primary reason for the variation in stress on the connecting rod under different spalling forms is that when the four-bar mechanism is coupled with the coal wall, the angle between the connecting rod is different, resulting in different bearing directions of the force, an increase in the angle, a decrease in the bearing capacity of the face guard mechanism, and an increase in the concentration of stress in the hinge hole.

### Analysis of the face guard mechanism’s force transmission properties under impact load

During the mining process of large mining height working faces, impact loads are generated on the hydraulic support due to the fracture of the overlying rock layer and the instability of the collapsed rock layer^39^. The occurrence conditions of coal seams are complex and variable, leading to variations in the flatness of the coal wall after mining by the shearer. This frequently leads to phenomena like the face guard only coming into touch with the coal wall in only one area. When there is poor contact between the face guard and the coal wall, and the impact load occurs, the hinge pins of the face guard mechanism are susceptible to impact fracture. Therefore, it is essential to research the face guard mechanism’s force transmission properties during various coupling states during impact loads.

**Figure 15:**
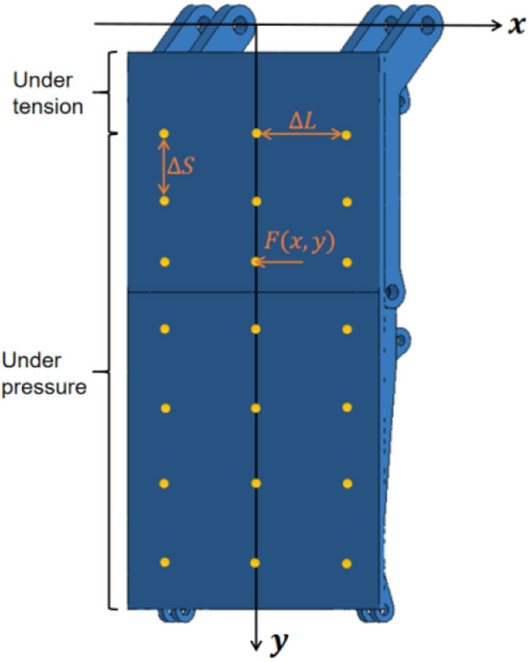
Schematic diagram of loading positions for impact loads on face guard.

Select the central axis of the hinge hole between the primary face guard and the extensible canopy as the x-axis, and the vertical line in the x-axis as the y-axis. Each interval along the x-axis direction $\Delta L$=550 mm, and each interval along the y-axis direction $\Delta S$=240 mm. A total of 21 action positions are taken to impact load the face guard. Only consider that the primary face guard is compressed when the impact load is applied to the face guard. Therefore, the impact load is applied at position ${y}_{d}$> 460 mm. Integrate the face guard mechanism’s bearing capacity under different forms of spalling, the impact force $F(x,y)$ is set to 50 kN to prevent the face guard jack from overflowing. As illustrated in Fig. 15, it is a schematic diagram of the impact load loading position for the middle-upper rib spalling face guard, the remaining forms of rib spalling all adopt this loading method.

**Figure 16:**
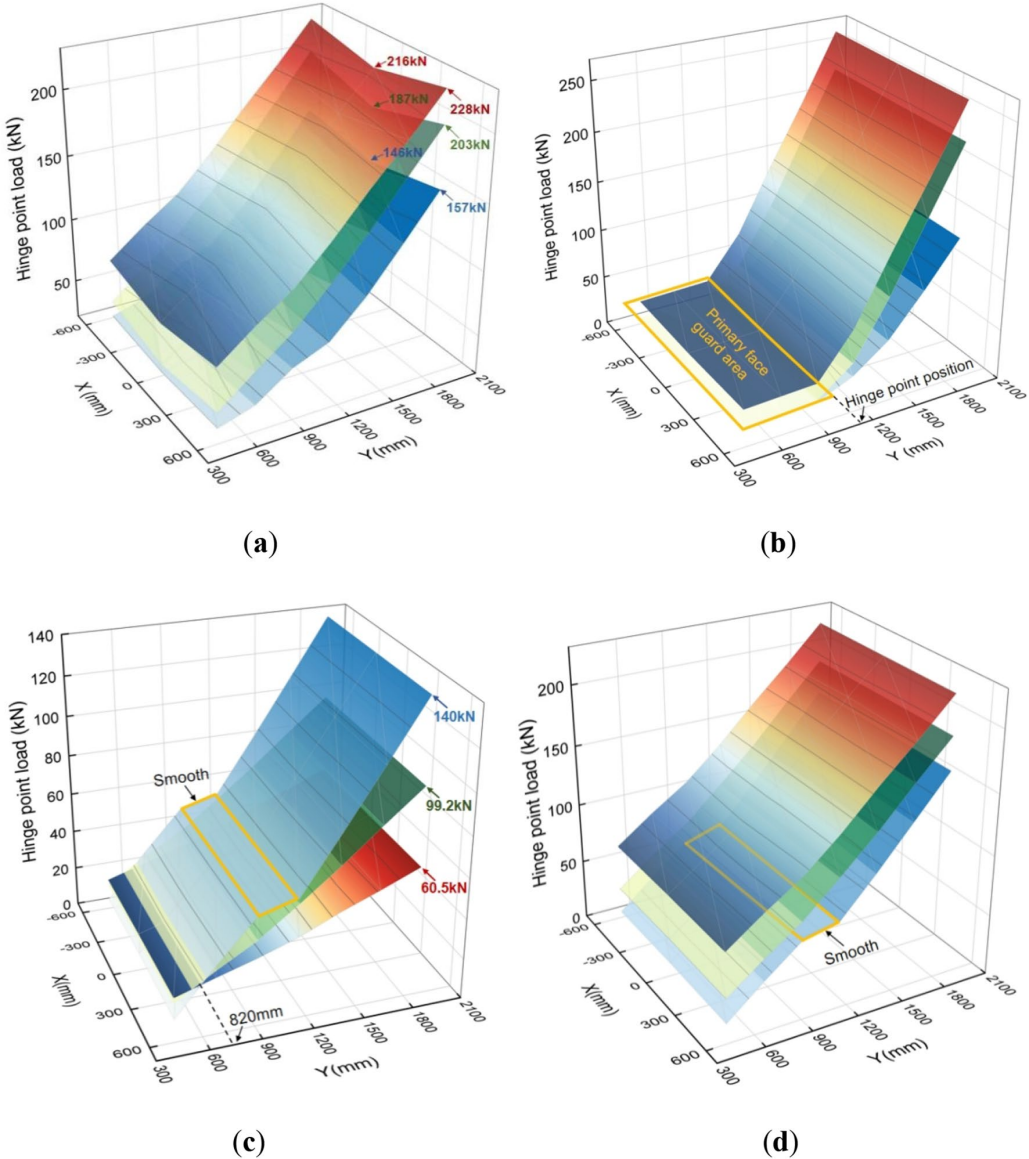
Comparison of force transfer characteristics in multiple coupled states under impact load: (**a**)the load at the hinge joint of the primary face guard—extensible canopy; (**b**) the load at the hinge joint of the primary face guard—secondary face guard; (**c**) the load at the hinge joint of the connecting rod 2—connecting rod 3; (**d**) the load at the hinge joint of the primary face guard—connecting rod 3.

The various hinge points of the face guard mechanism are critical locations for connecting components to transmit force. By analyzing and comparing the transmission characteristics of force at important hinge points under different forms of spalling, it can be beneficial to improve the load-bearing and protective capacity of the spalling. To more intuitively compare the force transfer properties of different coupling states during impact load, in this article the load data of the face guard mechanism carrying the impact load when the upper coal wall spalls is defined as a colored curved surface, the load data of the face guard mechanism under the impact load when the middle and upper coal wall spalls is represented as a green curved surface, while a blue curving surface represents the load data for the middle coal wall’s spalling. Figure 16 illustrates the force transfer properties of different coupling states during impact load.

Figure 16a illustrates the load change at the hinge joint of the primary face guard and extensible canopy. The load at the hinge point varies linearly along the support’s length direction under each of the three spall coupling states. On the other hand, the load at the hinge point tends to be greater on both sides and smaller in the center when the impact force moves in the support’s breadth direction, meaning that the load in the vertical direction of the face guard is smaller than the load on both sides. Also, the load difference at this hinge point reaches its maximum in each coupling state when the impact load applies upon both sides of the face guard’s end, indicating that the hinge point is highly sensitive to torque changes in the moment caused by the applied impact load’s location.

The load variation at the hinge joint, which is between the primary and secondary face guards, is depicted in Fig. 16b. The load at the hinge point hardly changes when the impact load is delivered to the primary face guard. Nevertheless, the stress at the hinge point increases quickly when the impact load is applied to the secondary face guard. In other words, the hinge point at which the primary and secondary face guards is only influenced by the impact load acting below its position, and the impact on the hinge point is most significant when coupled with the upper coal wall. The load variation at the connecting rods 2 and 3 hinge points under an impact load is shown in Fig. 16c. When the impact load moves along the length of the face guard and the position of action is less than 820 mm, the hinge point becomes more sensitive to the impact load response under the coupling state of the upper rib spalling. As the impact load position advances downward, the hinge point load resulting from the impact load in the coupled state of the upper and middle-upper rib spalling progressively increases. Among them, the hinge point load induced by the impact load in the coupled state of the middle rib spalling first increases, tends to be stabilize, and then rapidly rises again. Finally, the maximum load at the hinge point is 1.4 times that in the coupled state of the middle-upper rib spalling, and 2.3 times that in the coupled state of the upper rib spalling. The impact load response and static load response trends of the connecting rod 2-connecting rod 3 hinge points under different coupling states are basically consistent, and the stress situation is more severe when the hinge points are in the middle coal wall’s coupling state.

Figure 16d shows the load changes at the primary face guard and connecting rod 3’s hinge joint under impact load. The load change trend at the hinge joint of each spall coupling state is similar to that at the hinge joint where connecting rods 2 and 3 hinge together. However, when the impact load acts on the upper rib spalling coupling state and the middle rib spalling coupling state, their influence on the hinge joint load is opposite. According to the comprehensive analysis of Fig. 16c and d, it is found that the load changes in the middle region (940–1180 mm) of the connecting rod 2-connecting rod 3 hinge point and the primary face guard—connecting rod 3 hinge point under the coupling state of the middle rib spalling are relatively gentle. This is due to the coupling between the rib and the face guard during the middle rib spalling, resulting in a change in the direction of the force, which affects the force transmission in the region.

As inferred from the study of Fig. 16, the force transmission properties of the face guard mechanism’s hinge points depend on the impact load position and the coupling state of the rib spalling. When the face guard is coupled with the upper rib spalling, most of its hinge points are most sensitive to impact load (except for the connecting rods 2 and 3’s hinge locations), and the required bearing capacity of the hinge pin at the hinge point is greater. Compared with other forms of spall coupling, the face guard’s hinge point, in this state of spall coupling is more prone to wear, fracture, and other failure situations. Therefore, in the process of spall support, it is important to take into account various coupling states and the pin shaft’s bearing properties simultaneously to avoid damage and failure that may lead to safety accidents.

**Figure 17:**
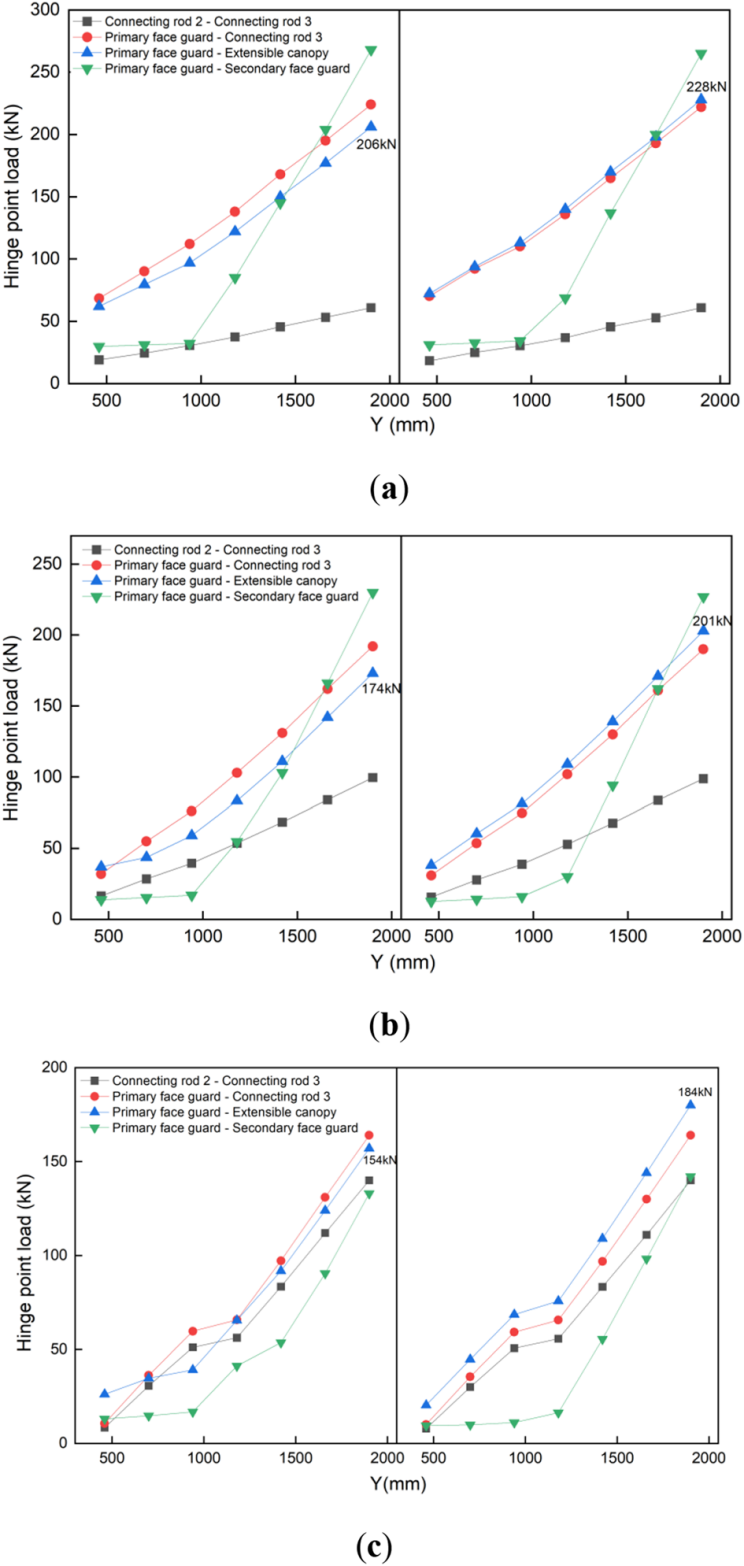
The force transfer characteristics of various spall coupling states under a biased load: (**a**) upper rib spalling; (**b**) middle-upper rib spalling; (**c**) middle rib spalling.

After the hydraulic support for large mining height changes from "small-scale and easily self stable" to "large-scale and easily unstable", the probability of being subjected to bias loads significantly increases^40^. When the face guard mechanism bears a biased load, its symmetrical hinge points bear differing transverse torques, consequently altering their mechanical characteristics. To investigate the impact of biased loads on the face guard mechanism’s hinge points, a contrasting analysis was conducted on the load variations of the symmetrical hinge points on both the right and left sides of the face guard during various spall coupling states when the impact load was applied on a straight line $x$=550 mm. Figure 17 illustrates the force transfer characteristics of various spall coupling states under a biased load. When the face guard mechanism experiences biased loads, the load variation trend of the key hinge points in each coupling state remains similar. Notably, the hinge position at the primary face guard and extensible canopy exhibits the highest sensitivity to the biased load position, this means that the torque change caused by the width variation significantly impacts the load at this hinge point. As the biased load position shifts downward, the primary face guard is hinged at the position of the extensible canopy, and the load disparity between both sides gradually amplifies. The maximum load disparity on the hinge point’s two sides under the three spall coupling states is recorded as 22 kN, 27 kN, and 30 kN, respectively. Simultaneously, when the secondary face guard experiences the biased load ($y>$ 940 mm), the load at the hinge point connecting the primary and secondary face guard under different coupling states, is also affected by varying degrees of transverse torque changes. The other hinge joints are not significantly affected by biased loads and exhibit good adaptability to such loads. Regardless of the coupling state between the rib spalling and the face guard mechanism, if the face guard mechanism is subjected to bias load for a long time, the gap between the hinge points on both sides of it will cause differential deformation and wear. Therefore, it is crucial to focus on the operational condition of the face guard mechanism under long-term eccentric loading, and promptly conduct inspections and repairs to avoid accidents caused by deteriorating support performance.

## Conclusions

To select a reasonable hydraulic support face guard mechanism, and improve the stability of the coal wall and support reliability. The mechanical models of the two structural forms of the integral and the split are established, and their structural characteristics and support differences are compared and analyzed. Theoretical analysis and rigid-flexible coupling numerical simulation are conducted on the coupling characteristics of the integral-type face guard mechanism’s rib spalling. The relevant conclusions were as follows:

1. The integral-type face guard mechanism outperforms the split-type in terms of load-bearing capability and effective support region. Under the same coal mining conditions, the integral-type is preferred.
2. Under various spall forms, the integral-type face guard mechanism’s bearing capacity varies in a similar way, and the bearing capacity decreases as the coal wall load position moves downward, but the supporting capacity of the primary face guard is quite different.
3. When the coal wall’s load is statically supported by the integral-type face guard mechanism, its response process can be defined in three stages: initial support, increasing resistance bearing, and constant resistance bearing, the stress concentration area of each stage is different. To meet support requirements, it is essential to appropriate reinforcement and maintenance in the stress concentration area.
4. The coupling state between the impact load position and rib spalling significantly influences the force transfer properties at each hinge points. The mechanism hinge joint is particularly sensitive to impact loads during the coupling state of the upper rib spalling. When compared to other forms of spall coupling, the hinge joint in this state is more prone to wear, fracture, and other failure situations.
5. When the integral-type face guard mechanism under different coupling states of rib spalls, experiences bias load, the hinge points at the primary face guard-extensible canopy exhibit the highest sensitivity to the position of the bias load, and this hinge joint is prone to differential deformation and wear. Other hinge points have better bias load adaptability.

In conclusion, the research results presented in this article contribute to the rational selection of face guard mechanism for the full-mechanized mining face with large mining heights and to carry out fault prevention. Furthermore, this study offers a more realistic, comprehensive, and intuitive studies of the coupling features between the coal wall and the face guard mechanism. This study can be used as a guide to regulate the face guard mechanism’s posture based on the recognition of coal walls. Nevertheless, this study has its limitations. While research has been conducted on the coupling characteristics of the main forms of rib spalling and protection mechanisms, the various forms of rib spalling are complex and diverse. For certain uncommon yet highly hazardous and difficult in governance forms of spall, the coupling characteristics between the coal wall structure and face guard mechanism remain unstudied. In future work, we aim to reveal the coupling characteristics of other rib spalling and conduct relevant experimental studies.

## Data Availability

The data used to support the findings of this study are included within the article.
